# An Overview of In Vitro Models of Alcohol‐Related Hepatocellular Carcinoma

**DOI:** 10.1002/cam4.70524

**Published:** 2025-01-16

**Authors:** Anoïsia Courtois, Sophie Gérard, Damien Esparteiro, Eric Nguyen‐Khac, Ingrid Marcq, Grégory Fouquet

**Affiliations:** ^1^ GRAP INSERM U1247, Curs Université Picardie Jules Verne Amiens France; ^2^ HGE département CHU Amiens Picardie Amiens France

**Keywords:** alcohol, hepatocellular carcinoma, in vitro models

## Abstract

**Background:**

Chronic and excessive alcohol consumption is the leading cause of death due to chronic liver disease. Alcohol‐related liver disease (ALD) encompasses a broad spectrum of clinical and pathological features, ranging from asymptomatic and reversible pathologies to hepatocellular carcinoma (HCC), a highly prevalent and deadly liver cancer. Indeed, alcohol consumption is one of the main worldwide etiologies of HCC. However, the impact of alcohol consumption on HCC pathophysiology and the associated mechanisms remains unclear. Thus, in vitro alcohol‐related HCC models are essential for addressing this issue and for assessing new molecular markers of this disease. In this review, we discuss the current in vitro models of alcohol‐related HCC. Our global overview demonstrates the lack of uniformity regarding HCC cell lines, alcohol concentration, and duration of alcohol exposure among existing models. Despite efforts to model alcohol exposure effectively that demonstrate enhancement of cancer cell transformation markers and HCC aggressiveness following, respectively, short‐term and long‐term alcohol exposure, current in vitro models possess numerous limitations.

**Aim:**

This review highlights future challenges in the development of more integrated and representative models of the complex pathophysiology of alcohol‐related HCC.

## 1 Introduction

### 1.1 Trends in Alcohol‐Related HCC

#### 1.1.1 Epidemiology

Approximately 4.1% of the population develops alcohol use disorders (AUD). Excessive and chronic alcohol consumption can lead alcohol‐related liver disease (ALD). The pathophysiology of ALD presents a broad‐spectrum of stages leading to the development of cirrhosis, the main risk factor of hepatocellular carcinoma (HCC). Indeed, fibrosis and cirrhosis are observed in 8%–20% of AUD patients [1]. Alcohol‐related cirrhosis is associated with an annual incidence of HCC of 2%–3% and enhances the 5‐ and 10‐year cumulative risk of HCC to 3% and 9%, respectively [2]. Due to the development of ALD, excessive and chronic alcohol consumption is one of the main etiologies of HCC, globally accounting for about 17.3% of HCC cases in 2020 and 245,000 deaths in 2015 [3]. The burden associated with alcohol is highly variable geographically: for instance, in Eastern Europe, alcohol is a contributing factor in up to 60% of HCC cases. Heavy drinkers have a relative risk of developing HCC of 2.07 compared to non‐drinkers. Furthermore, alcohol consumption can act synergistically with other HCC etiologies, such as hepatitis C virus (HCV) infection, resulting in a risk increase that can reach 53.9 [4]. Interestingly, alcohol cessation reduces the risk of developing HCC by 6%–7% per year, eventually reaching the same incidence of HCC as non‐drinkers after about 20 years of abstinence [5]. Despite some limitations, a French study demonstrated the benefits of diagnosis and management of alcohol use disorder in alcohol‐related cirrhosis, showing that such interventions influence patients' alcohol consumption and improve their prognosis. While there was no significant difference in median overall survival between non‐alcohol‐related HCC (15.7 months) and abstinent alcohol‐related HCC (11.7 months), the median survival was significantly shorter in non‐abstinent alcohol‐related HCC, with a median overall survival of 7.6 months, suggesting that alcohol‐related HCC patients would benefit from alcohol withdrawal [6]. However, a recent study reported that 34.9% of cancer patients continue to misuse alcohol, with 21% engaging in binge drinking [7]. In this context, understanding the pathophysiological mechanisms associated with the maintenance of alcohol consumption in patients suffering from HCC remains crucial.

### 1.2 General Aspects of Alcohol‐Related HCC

HCC is a complex multi‐step pathology characterized by significant intra‐ and inter‐tumor heterogeneity [8]. Despite biannual surveillance, many HCC cases are diagnosed at a late stage due to their asymptomatic development in the context of an underlying chronic liver disease. At diagnosis, regional lymph node involvement and metastasis are observed in 27% and 18% of HCC cases, respectively [9]. Transcriptomic analysis of HCC heterogeneity has led to the classification of HCC into two types: proliferative and non‐proliferative. Based on this classification, alcohol‐related HCC is defined as non‐proliferative, characterized by reduced aggressiveness and normal differentiation [10]. Several mechanisms associated with the pathogenesis of HCC induced by excessive and chronic alcohol consumption have been identified. Ethanol metabolism produces acetaldehyde, a mutagenic compound that leads to DNA repair failure, lipid peroxidation, mitochondrial damage, and various genetic and epigenetic alterations. Additionally, ethanol and acetaldehyde promote oxidative stress through the production of reactive oxygen species (ROS) in a cytochrome P450 2E1 (CYP2E1)‐dependent manner. ROS accumulation induces angiogenesis and metastatic processes [11]. Alcohol consumption also increases gut permeability and the translocation of pathogen‐associated molecular patterns (PAMPs) such as lipopolysaccharide (LPS). LPS activates Kupffer cells, leading to the production of pro‐inflammatory cytokines: this sustained inflammatory cascade promotes ALD progression and HCC development. Furthermore, alcohol misuse decreases the recruitment of CD8^+^ T cells, creating an immunosuppressive microenvironment and reducing the anti‐tumor immune response [4]. Despite these findings, the complete understanding and identification of all mechanisms by which alcohol consumption promotes HCC progression remains incomplete. To better elucidate these mechanisms, researchers have developed in vivo models using mice and rats. However, establishing in vivo liver cancer models in rodents is very challenging. Only 2% of rats develop adenomas, and a two‐year‐long alcohol consumption period is necessary to promote liver cancer [12]. Therefore, the development of simpler in vitro models is essential to simplify and elucidate the complex mechanisms underlying this cancerous development. In this review, we aim to provide an overview of the current in vitro models of alcohol‐related HCC, their limitations, and future challenges.

### 1.3 Current In Vitro Modeling of Alcohol‐Related HCC

The deleterious effects of ethanol have been studied in various in vitro models of HCC. However, each study has developed its own protocol for ethanol exposure, each with its unique characteristics, advantages, and limitations. These protocols vary significantly in terms of the cell lines used, as well as the duration and concentration of ethanol exposure. In this review, we have selected models that allow us to investigate the mechanisms of tumor transformation and cancer enhancement induced by ethanol. We define alcohol exposure up to a week as short‐term exposure, and alcohol exposure for more than a week as long‐term exposure. Tables 1 and 2 present the different in vitro HCC models of ethanol exposure.

**TABLE 1:** In vitro HCC models of short‐term ethanol exposure.

Cell line	EtOH concentration	Time	Alcohol mechanisms	References
HepG2, Hep3B	10–100 mM	24 h	↗ ERK1/2, ↗ cell growth, ↗ TGF‐α	[25]
HepG2, Hep3B	150 mM	16 h‐30 h	↗ IGFBP‐1 mRNA stability, ↗ JNK1/2	[27]
HepG2	20 mM	24 h	↗ ERK1, ↗ cell viability, ↗ Bcl‐2	[30]
HepG2	4,6% (787 mM)	24 h follow by 30 h recovery	↗ apoptosis, ↘ proliferation with alcohol; ↗ aggressiveness after recovery	[31]
HepG2	0.2% (44 mM)	24 h	↗ aggressiveness, ↗ protusions in co‐culture (HUVEC) 3D model, ↗ NF‐κB	[23]
HepG2, Huh‐7	100 mM	48 h	↗ FABP4 in CYP2E1‐expressed cells	[18]
HepG2	50 mM	2 h	↗ Brf1 MSK1‐dependent, ↗ cell proliferation, ↗ colony formation in soft‐agar	[17]
HepG2	25 mM	24 h, 48 h	↗ SIRT1 activity	[29]
HepG2	50 mM	1 h	↗ Brf1	[15]
HepG2	50 mM	1 h	↗ Brf1, ↗ RNA Pol III, JNK1‐dependent	[16]
Hep3B	50 mM	2–7 days	↗ SIRT7, ↗ CYP2E1, ↘ E‐cadherin	[28]

*Note:* Gray values represent the estimated ethanol concentration (in mM).

**TABLE 2:** In vitro HCC models of long‐term ethanol exposure.

Cell line	EtOH concentration	Time	Alcohol mechanisms	References
HepG2	75 mM	9 days	Global gene expression	[32]
Primary hepatocytes	100 mM	2 weeks	↗ CSC Markers, EMT, ↗oxidative stress	[33]
Hep3B	10 mM	40 passages	↗ LOX, ↗ aggressiveness	[34]
Hep3B	10 mM	40 passages	Cell cycle, ↗ mitomycin C resistance	[26]
Huh‐7, SNU449	80–270 mM	6 months	↗ aggressiveness, ↗ CSC markers, ↗ ERK1/2 and Akt pathways, withdrawal benefits	[20]
HepG2, SMMC‐7721	0.2% (44 mM)	2–3 weeks	↗ aggressiveness, EMT, ↗ spheroids formation	[22]

Abbreviations: CSC, cancerous stem cells; EMT, epithelial‐to‐mesenchymal transition.

#### 1.3.1 HCC Cell Lines and Culture Models

In the literature, various differences are noted depending on the models used. Firstly, the most common models are based on HCC cell lines cultured in a 2D monolayer and the most commonly used HCC cell line in studies of alcohol effect is the HepG2 hepatic cancer cell line (Tables 1 and 2). This cell line is non‐tumorigenic and contains a mutated alcohol dehydrogenase (ADH) enzyme, which prevents the oxidation of ethanol into acetaldehyde, a compound that is even more toxic and harmful than ethanol [13]. The use of this cell line does not model the overall effect of alcohol metabolism but allows for differentiating the effects of ethanol alone from those of acetaldehyde.

In the study of Donohue et al., three recombinant cell lines were developed to study the impact of alcohol metabolism on the toxic effects of ethanol, named VL‐17A (HepG2‐CYP2E1^+^/ADH^+^), E‐47 (HepG2‐CYP2E1^+^) and VA‐13 (HepG2‐ADH^+^). VL‐17A cells cleared ethanol from the medium at a similar rate to that of VA‐13 cells, indicating that both the clearance of ethanol and the generation of acetaldehyde were highly dependent on the expression of ADH. This was further supported by the finding that E‐47 cells, which express only CYP2E1, exhibited little to no capacity for either function [14]. These recombinant HepG2 cell lines, transfected to express ADH, enabled the study of alcohol metabolism in the context of cancer [15, 16]. Interestingly, while a 2 h exposure to 50 mM ethanol enhanced Brf1 expression (an important factor for cell transformation and tumorigenesis) in VA‐13 cells, no effects were observed in the conventional HepG2 cell line, suggesting the determinant role of alcohol metabolism in liver carcinogenesis [17]. Likewise, in E‐47 cells as well as Huh‐7‐CYP2E1^+^ cells, 100 mM ethanol for 48 h increased FABP4 production in a CYP2E1‐dependent manner, a soluble protein implicated in cancer development and progression [18].

The impact of the HCC stage on the effect of alcohol has also been studied. Marié et al. used two HCC cell lines: Huh‐7, an early‐grade hepatoma tumorigenic cell line without mutation in the ADH enzyme [19], and SNU449, a cell line of an advanced stage of HCC (grade II‐III/IV) also positive for the hepatitis B virus (HBV). In this study, SNU449 cells demonstrated a lower ADH activity than Huh‐7 cells. Moreover, long‐term alcohol exposure enhanced HCC aggressiveness and cancer stem cells (CSCs) markers only in Huh‐7 cells [20]. In early‐grade Huh‐7 cells, chronic alcohol exposure seems to enhance the acquisition of characteristics specific to advanced‐grade SNU449 cells.

Finally, Hep3B cells, a tumorigenic HCC cell line that is positive for HBV, are used to study viral infections that can lead to the development of liver cancer and have also been used in alcohol studies [21].

However, the conventional 2D monolayer models can be insufficient to analyze CSCs behavior and aggressiveness, and only a few studies have developed 3D cell culture model. Chen et al. cultured cells in matrigel, an extracellular matrix (ECM), to observe the ability of cells to produce tumors. This model demonstrated the enhancement of tumor sphere production, also known as spheroids, following a two‐week alcohol exposure [22]. Wang et al. developed co‐cultures of HepG2 HCC cells with endothelial cells (HUVEC) in a 3D culture model exposed to ethanol. Alcohol exposure enhanced angiogenesis through the formation of sprouts in this co‐culture model [23]. However, many other integrated culture models need to be developed to study the impact of alcohol exposure on liver cancer.

#### 1.3.2 Concentration and Duration of Alcohol Exposure

The studies that investigated the effects of alcohol exposure on HCC used a range of alcohol concentrations from 10 to 787 mM. Short‐term exposure models are more numerous, with ethanol concentrations ranging between 10 and 787 mM, and from 10 to 270 mM in chronic exposure models. A study investigating the cytotoxic effects of ethanol in HepG2 cells demonstrated that, after 24 h of exposure, ethanol decreased cell viability starting from a 0.6% (approximatively 130 mM) concentration [24]. An extrapolation of blood alcohol levels determined that 44 mM (0.2 mg/dL) is considered a physiological concentration [25]. However, compared to primary human hepatocytes, HCC cell lines and 2D culture conditions present numerous differences, such as in their alcohol metabolism and absence of an immune system. In this context, the use of higher concentrations than 44 mM observed in numerous studies seems relevant.

Furthermore, alcohol evaporation is rarely accounted for in the protocols described in the literature. Huang et al. detailed the use of a specific incubator with an ethanol‐saturated atmosphere to minimize and counteract this evaporation [26]. An environment saturated with ethanol vapor has also been used by Chen et al., with the addition of a change in culture medium every 3 days [22]. Marié et al. addressed this issue by daily adding 25% of the initial exposure dose to compensate for evaporation. The analysis of alcohol concentration in the culture supernatant confirmed its stability [20].

The duration of alcohol exposure described in the literature vary from acute, as short as 30 min, to very chronic, with exposure lasting up to 6 months. While short‐term ethanol exposure (up to a week) is mainly used to analyze the expression of cell transformation markers (Table 1), long‐term exposure models (more than a week) seem to be more representative for analyzing mechanisms of HCC aggressiveness (Table 2). Indeed, unlike short‐term acute exposure, chronic exposure allows for the modeling of the adaptive and plastic capabilities of cancer cells during frequent ethanol assaults. The carcinogenic effects of ethanol are generally associated with chronic exposure, as seen in alcohol‐related HCC patient. Therefore, chronic exposure is more representative of the consumption behavior found in HCC patients.

Magne et al. exposed HepG2 liver cells to ethanol at a concentration of 150 mM for only 30 min [27]. In acute exposure (Table 1), Lin et al. investigated the effect of a 2‐h exposure to 50 mM ethanol on the HepG2 HCC cell line [17]. Other studies have modeled the impact of longer exposure, ranging from several hours to several days [28, 29, 30, 31]. Attal et al. exposed Huh‐7 and HepG2 cells to 100 mM ethanol for 48 h [18]. More chronic alcohol exposures, ranging from 1 week to 6 months, have been described in several studies [20, 22, 26, 32, 33, 34] (Table 2). Among them, Yu et al. studied the effect of exposure to 100 mM ethanol for 14 days on liver damage focusing on oxidative stress and CSCs [33]. The most chronic exposures found to date extend up to 40 passages and 6 months of exposure. Huang et al. exposed their Hep3B cells to 10 mM ethanol for up to 40 passages [26]. Marié et al. exposed Huh‐7 and SNU449 cells to different concentrations of ethanol ranging from 80 mM to 270 mM daily for 6 months [20]. This model allowed the study of the impact of alcohol on the aggressiveness of HCC, focusing on invasion, migration mechanisms, and CSCs.

Management of alcohol addiction in order to achieve abstinence is the best care in the case of cirrhosis (90% of HCC cases) to improve overall survival. However, only one study has analyzed the molecular and biological benefits of in vitro alcohol withdrawal after long‐term alcohol exposure. Marié et al. demonstrated that, following exposure to 80, 160, and 270 mM ethanol for 6 months, a one‐month withdrawal resulted in a notable partial reduction of alcohol effects in the Huh‐7 HCC cell line [20]. Indeed, alcohol withdrawal resulted in the reversal of alcohol‐induced aggressiveness mechanisms, including cell migration, invasion, and CSCs markers such as CD133. This study demonstrated that withdrawal has beneficial effects on the HCC aggressiveness induced by alcohol.

### 1.4 Limitations of Existing In Vitro Alcohol‐Related HCC Models

Currently, the impact of alcohol exposure is mainly studied on 2D monolayer cell culture. Indeed, this technique is easy‐to‐use, cost‐efficient and allows for the characterization of cellular and molecular mechanisms, alcohol metabolism and drug toxicity [35]. The most commonly used models, HCC cell lines, are well‐characterized with specific alcohol metabolism but are limited by a lack of genetic and epigenetic heterogeneity. The use of primary hepatocytes, the gold‐standard from human or mouse, can partially address these limitations. However, primary hepatocytes rapidly dedifferentiate in 2D monolayer cell cultures leading to the loss of hepatocyte functions [17, 28]. Therefore, in the context of alcohol‐related carcinogenesis, where long‐term alcohol exposure remains essential, these models are limited.

Understanding the complexity and mechanisms of alcohol‐related HCC pathophysiology requires cell–cell and cell‐ECM interactions. For example, liver injury activates the immune system, including Kupffer cells, and hepatic stellate cells (HSCs) that produce ECM. Thus, the development of co‐culture models remains crucial. In this sense, Wang et al., conducted a co‐culture study with HepG2 cells and endothelial cells (HUVEC) on a fibrin gel bead to determine the impact of alcohol on angiogenesis [23]. Despite its advantages, this model remains insufficient due to a lack of other cell types, such as cancer‐associated fibroblasts or tumor‐associated macrophages, which promote angiogenesis through the production of vascular factors [36]. In addition, ECM plays an important role in the loss of hepatocyte polarity, leading to epithelial‐to‐mesenchymal transition [37]. The absence of ECM in the current models presents some limitations in understanding the pathophysiology of alcohol‐related HCC.

Another particularity of HCC is the heterogeneity of the tumoral hepatocyte population. Indeed, in the core of the bulk tumor, CSCs are selected in a restrictive and poor microenvironment generated by oxygen and nutrient deprivation, the presence of necrotic bodies, and an acidic microenvironment [38, 39]. Currently, 2D monolayers or spheroids studies are unable to generate this heterogeneous microenvironment. Chronic alcohol exposure favors the development of CSCs as previously described in the liver [20, 39] through the Nanog‐TLR4 pathway, which enhances the formation of tumorospheres [40].

In vitro HCC models are often limited in their ability to accurately replicate the complexity of the disease in patients. Nevertheless, some models allow for the study of metabolism and HCC aggressiveness using markers such as CD133 [20], which is also present in patients. It is also possible to study other mechanisms, such as the microenvironment, using co‐culture models [23]. However, the results obtained in vitro may not always be directly translatable to clinical contexts and do not represent a fully integrated system. They lack complex interactions, such as those between tumor cells and the immune system. This underscores the importance of developing more integrated models, such as in vivo models, to better understand HCC pathology and develop effective treatments.

### 1.5 Challenges and Future Directions for In Vitro Alcohol‐Related HCC Models

Understanding the pathophysiology of alcohol‐related HCC remains challenging due to the limitations of the 2D cell culture models. The current challenge lies in developing more integrative and complex models to address numerous questions, such as the underlying cell mechanisms and the best therapy within the context of personalized medicine. The use of 3D cultures, such as organoids or tumoroids, and microfluidic devices seem to be a good compromise. These cultures are derived from various types of pluripotent stem cells and maintain properties including self‐organization, self‐renewal, differentiation, histological characteristics, genetic heterogeneity, and HCC tumor markers [41]. These techniques enable the overcoming of some of the limitations of 2D culture, such as the spatial organization of cells and the complexity of cell–cell and cell‐microenvironment interactions, mimicking organ structure and function [42].

In the context of ALD, the use of HepaRG spheroids has already been established and provides a better understanding of alcohol toxicity and steatosis but not of the cancer mechanism. However, this model only considers one cell type. The addition of other cell types, such as macrophages and HSCs, has already been performed by Bronsard et al. to study non‐alcoholic fatty liver disease, adding mechanisms of fibrosis and inflammation [43]. This model deserves to be studied in the context of alcohol exposure. The HepaRG model, used as a gold standard for liver cell toxicity and metabolism, is based on a non‐cancerous cell line and presents some limitations for studying HCC. The development of patient‐derived HCC organoids (PDOs) which preserve the molecular features of the original tumor, makes these models a more accurate route to study tumor development, and can be an interesting solution [44]. Indeed, PDOs from tumor needle biopsies represent an integrative and personalized model with histopathological, genetic, and molecular heterogeneity, a large panel of cell types, and the ability to remodel cell‐ECM interactions [45]. Interestingly, the mean age of HCC patients is about 65 years old suggesting the impact of aging on cancer development. In this context, PDOs offers to follow the impact of many factors associated to the development of HCC such as age or the natural history of alcohol consumption. In recent years, taking etiology into account, such as alcohol, for the best HCC therapy has become a significant focus, and in the future decades, PDOs could be a crucial model for personalized cancer modeling and drug screening.

Interestingly, PDOs can also reflect genetic heterogeneity. We have previously described the impact of ALDH and ADH, which are frequently associated with mutations such as ALDH2*2 and ADH3*1, in AUD and liver carcinogenesis. However other genes that predispose individuals to HCC deserve particular attention, such as PNPLA3. Indeed, the PNPLA3 Met148 variant (148 M) is associated with an increased risk of cirrhosis, up to 3.37‐fold in ALD patients, leading to HCC [46]. The impact of the 148 M variant of PNPLA3 has been studied in HCC cell lines using 2D culture, which has provided valuable insights into the critical role of this mutation in liver disease. HCC cell lines overexpressing the 148 M mutation has already been used, demonstrating alterations in lipid and glucose metabolism, but the role of this mutation has yet to be studied in the context of alcohol exposure [47]. In the era of molecular biology, the challenge is to identify and understand the role of these variants in the pathophysiology of alcohol‐related HCC to determine new therapeutic strategies. Other genetic variants, such as TM6SF2 (E167K), GCKR (P446L), and MBOAT7 (rs641738), also warrant closer investigation.

Despite their numerous advantages, organoids present several limitations that need to be considered: the protocols for organoid or tumoroid formation are numerous and variable, complicating the reproducibility of experiments, and some parameters remain difficult to control, such as the establishment of stable and complete vascularization and infiltration by immune cells [48]. Finally, although these models are complex, they do not allow us to capture systemic interactions and therefore cannot provide a complete and dynamic view of the alcohol‐related HCC mechanisms.

Microfluidic chips are innovative techniques that combine technology and engineering, overcoming some limitations. This technique facilitates the study of single cells and the creation of complex co‐cultures and organoids, which is important considering the cell–cell and cell‐microenvironment interactions in the liver. These microfluidic systems offer complete control over the composition of the microenvironment, including nutrient concentrations and oxygenation gradients. The use of flow media in these devices also makes it possible to reproduce physiological constraints, such as shear stress generated by the pressure of the surrounding flows, gas and nutrient diffusion, and waste elimination [49]. Moreover, given that the liver is a highly vascularized organ for its detoxification function, it is crucial to take this aspect into account. Chips enable the establishment of stable and dynamic vascular systems, allowing the study of interactions between hepatic microtissues and endothelial cells and potentially enabling the study of angiogenesis in the context of alcohol‐related HCC [50]. However, while these technologies are currently being improved, several limitations remain to be addressed. Indeed, these techniques are very expensive, and their use is complex, requiring numerous physical and technological parameters to be considered, such as fluid manipulation and behavior within the chip, as well as the various cellular stresses generated by flow pressure or the chip itself.

The use of microfluidic chips, which can enable real‐time study of cell behavior in the context of alcohol exposure, is a viable option and has already been demonstrated. These chips can model the impact of alcohol exposure on complex co‐cultures and study the resulting toxicity, metabolic changes, and genotoxicity [51]. Perfusion of a clinically relevant alcohol concentration enhances steatosis and modifies the expression of gene implicated in alcohol metabolism. This model also allows the perfusion of bacterial endotoxin, which is observed in the case of alcohol‐related HCC due to gut microbiota dysbiosis and increased gut permeability [52]. A future challenge is the development of multi‐organs‐on‐chip to enhance the in vitro understanding of the alcohol‐related HCC pathophysiology.

Integrating 3D cultures like organoids and tumoroids with microfluidic technologies shows potential in the in vitro research of alcohol‐related HCC. This approach allows us to move beyond the limitations of traditional 2D monolayer models and progress towards more integrated experimental systems.

## 2 Conclusion

In this review, we describe the current in vitro culture models that demonstrate the cellular and molecular mechanisms induced by alcohol exposure in HCC. Developing in vitro HCC models is crucial to enhance our understanding of alcohol‐related HCC mechanisms and improve patient management options. In accordance, the deleterious effects of alcohol have been studied in numerous cellular models. However, the lack of uniformity in concentrations and durations of alcohol exposure has limited the comparison between studies. The interindividual heterogeneity of HCC, liver metabolism, the various multicellular types of the liver, and the interactions with other organs make understanding the mechanisms of alcohol in HCC very complex. Cancer models are continuously evolving, and choosing an appropriate and universal model of alcohol exposure that best imitates the given HCC entity is a significant challenge for researchers. A promising perspective is the combination of PDO models with multi‐organ‐on‐chip systems, which represents the best compromise to capture the complexity and heterogeneity of HCC.

## Author Contributions

**Anoïsia Courtois:** investigation (supporting), writing – original draft (equal), writing – review and editing (equal). **Sophie Gérard:** investigation (supporting), writing – original draft (supporting). **Damien Esparteiro:** writing – review and editing (supporting). **Eric Nguyen‐Khac:** writing – review and editing (supporting). **Ingrid Marcq:** investigation (equal), writing – original draft (equal), writing – review and editing (lead). **Grégory Fouquet:** investigation (lead), writing – original draft (equal), writing – review and editing (lead).

## Conflicts of Interest

The authors declare no conflicts of interest.

## Data Availability

Data sharing is not applicable to this article as no new data were created or analyzed in this study.
